# Anthropogenic impact on soils of protected areas—example of PAHs

**DOI:** 10.1038/s41598-023-28726-6

**Published:** 2023-01-27

**Authors:** Alicja Kicińska, Piotr Dmytrowski

**Affiliations:** 1 Faculty of Geology, Geophysics and Environmental Protection, Department of Environmental Protection, AGH University of Science and Technology, Mickiewicza 30 Av, 30-059 Kraków, Poland; 2 Landscape Parks of the Małopolska Region, Vetulaniego 1A, 31-227 Kraków, Poland

**Keywords:** Environmental sciences, Environmental social sciences

## Abstract

The following study presents the concentrations of 10 individual polycyclic aromatic hydrocarbons (PAHs_10_) and the total concentration of PAHs (ΣPAHs) determined in soils of protected areas in Southern Poland (EU). The protected areas discussed here include 5 landscape parks and 5 nature reserves located in the Małopolska region. Surface soil samples were collected at 39 locations characterised by different natural conditions and different human pressure level. The samples were used to determine the contents of anthracene, benzo[a]pyrene, benz[a]anthracene, benzo[b]fluoranthene, benzo[k]fluoranthene, benzo[g,h,i]perylene, chrysene, dibenz[a,h]anthracene, indeno[1,2,3-cd]pyrene and naphthalene. These concentrations of individual PAHs ranged from < 0.005 to 6.34 mg/kg. When considering the legal regulations currently in force, this meant that permissible values were exceeded in 23% of the samples analysed, while increased concentrations were found in another 26% of the samples. The limit values were exceeded most in the case of benzo[b]fluoranthene and benzo[a]pyrene. This occurred with samples collected in the vicinity of transportation routes, mainly local roads. In the case of naphthalene and anthracene, the limit values were not exceeded. Increased or excessive PAHs concentrations do not occur in the vicinity of major industrial plants located near the boundaries of individual landscape parks, which indicates how these pollutants spread. The paper also points to the need to develop new legal solutions to improve the method for assessing PAHs concentrations and their impact on valuable natural areas.

## PAHs in the environment: introduction—studies of PAHs in landscape parks

Polycyclic aromatic hydrocarbons (PAHs) have been the subject of numerous studies due to their common occurrence as well as genotoxic, mutagenic and carcinogenic properties^1–5^. PAHs are a group of several hundred chemical compounds with various structural forms^6^. Most of these compounds are hydrophobic organic pollutants^2,7^. They are present in natural fuel deposits and they are formed as a result of burning coal, pyrolysis of fossil fuels and incomplete combustion of organic compounds. Their presence in considerable amounts can be a potential hazard to human health and life^8–11^. Due to their widespread occurrence in almost all components of the environment, including soils, PAHs are a considerable hazard to agriculture^12^. Also, despite their low water solubility, low volatility and hydrophobic properties, they have the ability to penetrate into the trophic chain^4,13–15^. The intake of these substances by mammals and humans causes changes in cytochrome enzymes found in the liver, kidneys, gastrointestinal tract and also in the lungs. Some PAHs are classified as most harmful pollutants to human health due to their strong toxic, carcinogenic, mutagenic and teratogenic properties. They are especially dangerous in highly urbanised areas located near legally protected natural areas^16^. The evaluation of the non-agricultural environment, especially that of protected natural areas, comprising cyclical monitoring of its individual components, is rarely conducted^17–20^. It is especially rare in the case of forms of protection such as landscape parks that protect both nature and the landscape and often contain very attractive and valuable natural forms. At the same time, such areas are subject to considerable human pressure, especially that caused by increased vehicle traffic, industrial operations (often including centuries-long traditions of mining and processing of minerals) and ongoing urbanisation.

Polycyclic aromatic hydrocarbons are a group comprising several hundred hydrocarbons characterised by a different relative position of two or more benzene rings in their molecule^2,13^. The half-life values of PAHs in the environment depend on their physical and chemical parameters and range from several hours to several days when in contact with atmospheric air, or up to several dozen years when confined in soil^21–23^. Owing to their toxic properties and their propagation in the environment as pollutants, 16 PAHs were listed as priority pollutants in the 1970s. Since then, they have been covered by monitoring studies worldwide^24^. The most commonly studied PAHs include^25–28^: acenaphthylene (Acy), acenaphthene (Ace), anthracene (Ant), benzo[a]pyrene (BaP), benzo[e]pyrene (BeP), benz[a]anthracene (BaA), benzo[b]fluoranthene (BbF), benzo[k]fluoranthene (BkF), benzo[g,h,i]perylene (BghiP), chrysene (Chr), dibenz[a,h]anthracene (DahA), fluorine (Flu), phenanthrene (Fen), fluoranthene (Fth), indeno[1,2,3-cd]pyrene (IndPir), naphthalene (Naf) and pyrene (Pir). These compounds are never found individually in the environment, they always appear in mixtures^29^. Environmental studies confirm that if one of the PAHs is found in a sample, other compounds belonging to this group are also present^30^. The main source of polycyclic aromatic hydrocarbons are fossil fuels i.e. coal and oil^31^. PAHs are released during the combustion of these fuels for municipal purposes (e.g. in power plants and CHP plants) as well as in individual households. Production processes involving fossil fuels are also a source of PAHs^32,33^. Another source is transport and emissions from various vehicles^1,24^. Individual exposure is caused by smoking tobacco and some food preparation methods e.g. smoking and grilling. Polycyclic aromatic hydrocarbons may also be released naturally into the environment as a result of e.g. volcanic activity or forest fires^26,34^. In conjunction with water vapour suspended in air, PAHs become components of smog. They can also contaminate soil and water, especially surface water^27,35^. PAHs indicating high bioavailability and bioaccessibility can easily enter plants from soil and water through their roots and bulbs (which are also consumed by humans), as well as being taken in from the air (e.g. through leaves and needles)^15,18–20,36,37^.

PAHs are found in almost every aspect of the environment, including plants, air and soil and, as a result, they can enter food products^38^. Because of this, exposure to PAHs is continuous and intensive^39,40^. One of the effects some PAHs have on the human body is the initiation of carcinogenesis^41–44^. The substance causing the most severe carcinogenic effects is benzo[a]pyrene^14,45^. It has been classified by the International Agency for Research on Cancer as carcinogenic to humans. There is no exposure threshold, which means that exposure to any concentration of this substance can result in cancer. Polycyclic aromatic hydrocarbons are also associated with the risk of premature birth and foetal development defects^14,15^.

In Poland, 83.7% of PAHs emissions are caused by non-industrial combustion processes (Table 1). These are mostly low emissions related to the combustion of fuel for purposes of individual household heating^46^. This causes considerable seasonal variation of emissions^47,48^. Production processes are responsible for a relatively high share of PAHs emissions (9.63%), followed by waste management (2.55%), agriculture (2.12%) and road transport (0.94%).

**Table 1:** Estimated annual PAH emission in Poland in 2017, including emission sources (according: KOBiZE, 2019).

Categories of emission sources	PAH	
	[Mg]	[%]
Combustion processes outside industry	126.87	83.70
Production processes	14.60	9.63
Waste management	3.87	2.55
Agriculture	3.21	2.12
Transport	1.43	0.94
Combustion processes in industry	0.83	0.55
Other vehicles and devices	0.61	0.40
Combustion in the energy production and transformation sector	0.14	0.10
The use of solvents and other products	0.01	0.01
Overall	151.58	100

Polish and European legal regulations specify several forms of nature protection, for example, national parks, nature reserves, landscape parks, natural and scenic complex, ecological areas, natural monuments, documentation sites and species protection. In general, these can be divided into protected areas and protected sites. Every type of protected area has different characteristics. National parks and nature reserves usually provide strict protection and are made available to members of the public to a limited extent. To fulfil their protective function these areas are not subject to urbanisation and large-scale human pressure. However, landscape parks are a different form of protection. Polish law defines these as areas protected due to their environmental, cultural, historic and landscape values^49^. Despite their protected status, they can be used for various forms of economic activity, but only in a sustainable way.

At present, there are 126 landscape parks in Poland. Their total area is 26,142 km^2^, which amounts to 8.36% of the total area of the country^50^. So far, there have been no systematic studies or monitoring of xenobiotic pollution, including PAHs concentrations, in the soils of protected areas in Poland, even more so in landscape parks. The existing data from the national network monitoring the presence of PAHs (usually BaP) in the environment apply mostly to air quality^47,51^.

Thus, taking the above into consideration, the present study aims to: (*i*) collect research material in the form of surface soil samples from 5 landscape parks and 5 nature reserves located in the Małopolska region; (*ii*) establish the concentrations of the 10 most prevalent PAHs; (*iii*) assess the level of soil contamination with PAHs in the protected areas studied and (*iv*) formulate recommendations related to the methods and frequency of monitoring appropriate for this type of hazard.

The goals presented in items 1 through 4 are innovative, because no studies discussing such issues in relation to protected areas in Poland have been conducted and published to date.

## Materials and methods

### Research area

**Figure 1:**
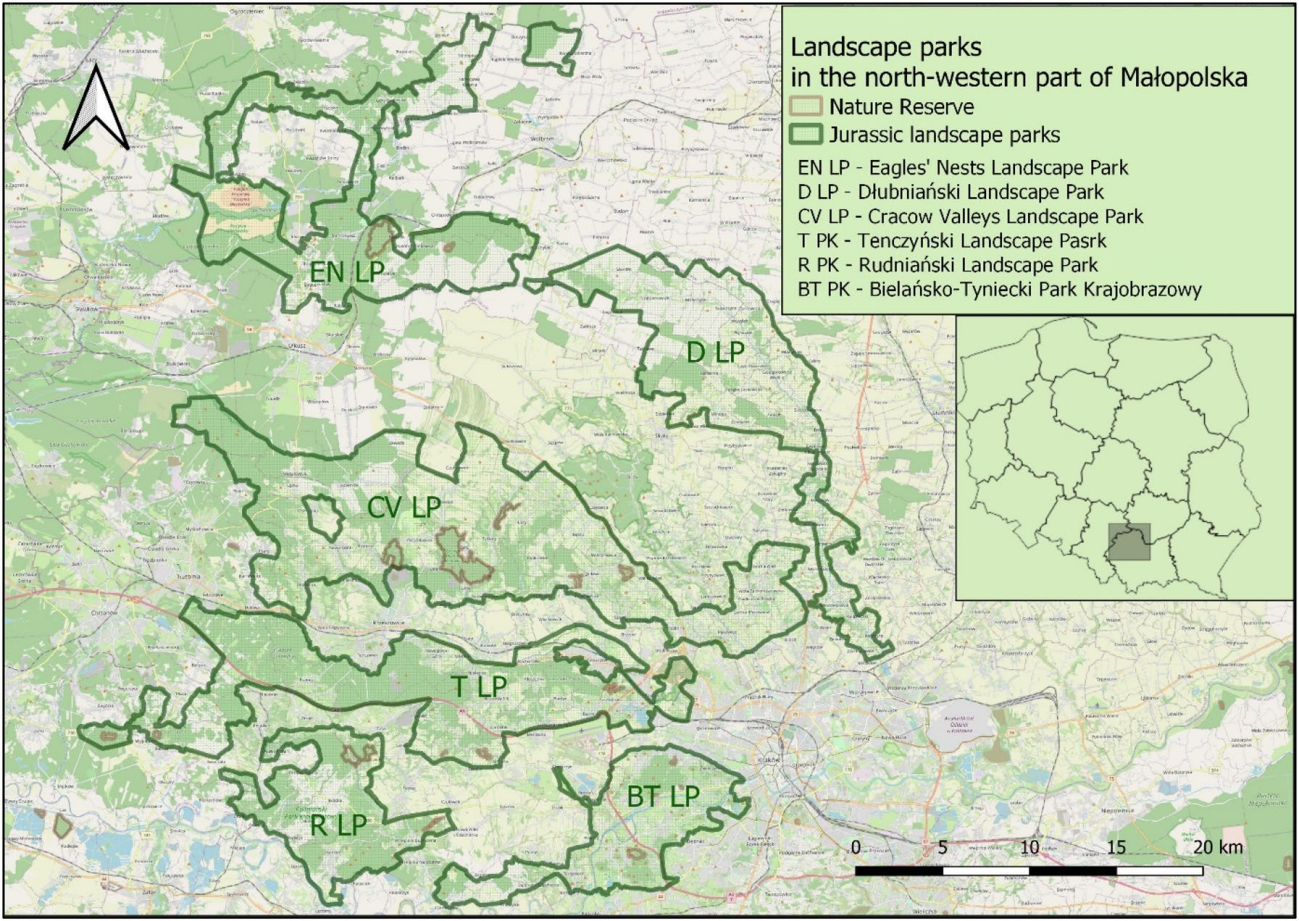
Location of landscape parks (LP) in South Poland (OpenStreetMap).

The study was conducted in 5 landscape parks (LP) located in the Małopolska region (Southern Poland, EU): Bielańsko-Tyniecki LP, Dolinki Krakowskie LP, Orlich Gniazd LP, Rudniański LP and Tenczyński LP (Fig. 1). The presence of different types of soil in the specified areas depends mostly on the type of bedrock that is the foundation for soil-forming processes. In the case of Małopolska, the most prevalent types include carbonate rock, limestone (formed as a result of sedimentation processes) and loess (formed as a result of Aeolian processes). The dominant soil types are brown rendzina soil, brown soil, aquic hapludalf and podsolic soil. Chernozem soils are much less common, while alluvial soil is found in river valleys^51^. Rendzina soil is one of the soils formed from calcareous carbonate rocks. It is characterised by a high content of rock fragments. The thickness of the soil profiles ranges from several tens of centimetres to over a metre. A characteristic feature of these soils is the brown horizon (Bw), which is formed under the humus horizon A, meeting the criteria of the diagnostic cambic horizon. Rendzina soils are rich in calcium, sometimes also in magnesium (especially when dolomites are present in the subsoil). In these soils, humus-mineral complexes are formed, and iron and aluminium compounds are present in small quantities and do not migrate from the surface to the deeper layers of the profile. The predominant reaction is neutral or alkaline, but the surface horizons, especially the A horizon, can be acidic or weakly acidic. The agricultural potential of the rendzina may be limited by its skeletal nature and its susceptibility to drying out, despite its predominantly loamy texture (typical record of the brown rendzinas profile: Ap-Bwca-Cca-Rca). When it comes to valuation class, the soils in the study area usually belong to class III or IV. The dominant morphological forms in Małopolska are highlands and mountains and the climate is temperate.

Potential sources of PAHs in this region include transportation routes and vehicle traffic along these routes, as well as low emissions. The transportation routes in the area discussed form a very dense network and include various categories of roads from motorways, through national roads and province roads, to commune roads. The traffic is very heavy and variable. The second important source of PAHs are low emissions, in most cases related to the combustion of fuels (mostly coal) and burning of various types of waste. Thanks to a number of social and government initiatives, the latter is becoming less of an issue, but environmental reports indicate that the pace of this ongoing change is still insufficient^52,53^. Potential emitters of PAHs in Małopolska may also include industrial plants located near the boundaries of the landscape parks studied, i.e. the Siersza Power Plant, the chemical plant in Alwernia and the mining and smelting plant in Bukownia. This group may also include large rock mining plants in Dubie, Czatkowice, Tenczynek and Zalas. Additionally, the concentration of pollutants in soils may be related to historic mining plants in Nielepice, Miękinia, Nowa Góra, Dębnik, Zakrzówek and Mirów, which are no longer in operation^47,51,52^.

### Soil sampling

Surface soil samples were collected in 5 landscape parks in the Małopolska region (Fig. 1) in the years 2020–2021 to determine the concentration of PAHs. A total of 39 soil samples were collected (Table 2). The majority of samples were collected in close proximity (1–3 m) to transportation routes (*n* = *31*) or other potential pollution emitters (operational quarries, industrial plants etc.) (Table 2). Samples were collected in areas with various forms of land use. The most common among them were forest areas (*23*), agricultural areas (*8)* and unmanaged greenery areas (*6*). Some samples were also taken at protected areas designated as nature reserves (*5*). Nature reserves in Poland are more strictly protected areas, where human activity should be kept to a minimum^53^.

**Table 2:** The number of soil samples and their collection sites.

Sample from		Landscape Park					Total
		Bielańsko-Tyniecki	Tenczyński	Dolinki Krakowskie	Orlich Gniazd	Rudniański	
		*10*	*10*	*9*	*5*	*5*	*39*
		*n*					
*Division according to use*							
1.1	Forest area	4	5	8	4	2	23
1.2	Agricultural area	4	2	–	–	2	8
1.3	Unmanaged greenery area	2	2	1	1	–	6
1.4	Other		1	–	–	1	2
*Division according to*							
2.1	Nature reserves	–	–	3	1	1	5
2.2	Near traffic	10	7	6	4	4	31
2.3	Industry influence	–	3	–	–	–	3

Each separate sample consisted of 5 batches of soil (weighing about 250 g each) excavated from a depth of 0–20 cm. Batches were taken from the corners and from the centre of a 1 × 1 m square. In total, about 1 kg of soil was taken at each location. Samples were stored and transported to a laboratory in dark glass containers at a temperature of ± 5 °C. Samples were pre-processed and analysed in a laboratory^54^.

### PAH determinations

PAH concentrations were established after performing a double liquid–solid extraction using *n*-hexane. The extraction was performed in the presence of the Semi-Volatiles Internal Standard Mixture (Restek 31,006). The resulting extract was dried with anhydrous sodium sulphate and filtrated through a column with silica gel. The aliphatic hydrocarbon fractions were eluted with hexane, while PAHs were leached with dichloromethane and concentrated to a volume of 1 ml. In the final phase, the 2-fluorobiphenyl standard (Restek 31,091) was added. The concentrations of naphthalene (Naf), acenaphthylene (Acy), acenaphthene (Ace), fluorine (Flu), phenanthrene (Fen), anthracene (Ant), fluoranthene (Fth), pyrene (Pir), benz[a]anthracene (BaA), chrysene (Chr), benzo[b]fluoranthene (BbF), benzo[k]fluoranthene (BkF), benzo[a]fluoranthene (BaF), benzo[a]pyrene (BaP), benzo[e]pyrene (BeP), indeno[1,2,3-cd]pyrene (IndPir), dibenz[a,h]anthracene (DahA), benzo[g,h,i]perylene (BghiP) and pyrene (Pir) in the extracts were determined using an Agilent Technologies 7890B gas chromatograph combined with an MSD 5977B mass spectrometer. The analysis involved using a Select PAH (Agilent CP7461) capillary column with a length of 15 m, a diameter of 0.15 mm and a film thickness of 0.1 µm. The results obtained were corrected for recovery based on the internal standard used. The limit of quantitation for each of the determinations was 0.005 µg/dm^3^ and the total PAH concentration was the sum of all the individual compounds. The determinations were performed at an accredited laboratory (accreditation no. AB 918).

Statistical calculations were conducted using Statistica ver. 13.3 and Excel. The differences between means were detected with Tukey’s HSD test at a significance level of 0.05.

## Results and discussion

**Figure 2:**
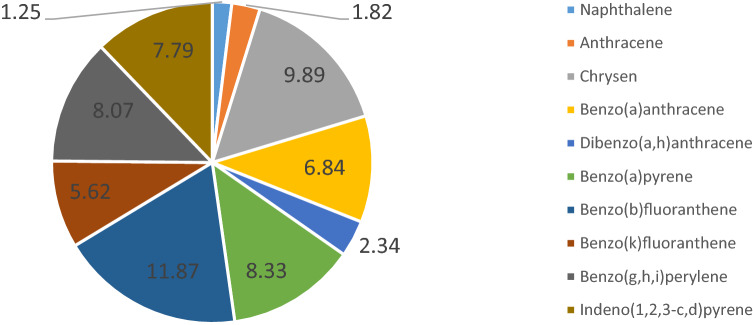
Share [%] of individual hydrocarbons in the total PAHs determined in soils from protected areas.

The concentration of specific PAHs ranged from < 0.005 to 6.340 mg/kg, while the total concentration of PAHs (ΣPAHs) in each of the 39 samples analysed ranged from < 0.090 to 36.300 mg/kg (Table 3). Determinations below the method's limit of quantitation amounted to 13% of all the results. Determinations ranging from 0.005 to 0.1 mg/kg (a legal limit value set for agricultural and green areas) amounted to 61% of the results. Concentrations of PAHs exceeding 0.1 mg/kg amounted to almost 26% of all the results obtained using the material analysed. Bojakowska and Sokołowska^55^ state that the concentration of PAHs in soils in Poland ranges from 0.001 mg/kg (in areas located far from industrial centres and not used for agriculture) to several thousand mg/kg in industrial areas such as refineries. Furthermore, Maliszewska-Kordybach^56^ reports that the mean concentration of PAHs is 0.327 mg/kg and depends on the soil properties, the most important being the content of organic substances^57^. The soil in the area analysed contains 2.40–6.22% humus and 1.39–3.61% organic carbon. When comparing the median total concentration of ΣPAHs calculated for all the samples collected (amounting to 0.761 mg/kg) to the median value calculated for 216 measurement-control sites located in arable land throughout Poland (amounting to 0.191 mg/kg), it can be said that it is higher than the mean concentration^58^. Similar conclusions can be drawn when comparing the results obtained to the monitoring data gathered in the period 1995–2020 at a site in Czajowice (Wielka Wieś Commune, Krakowski District, Małopolskie Province). It was found that the results are over 10 times higher than the mean concentration of 13 PAHs determined in the soil at that site in the year 2020 (0.064 mg/kg). The concentrations of individual hydrocarbons presented in descending order were as follows (data calculated for *n* = *39*, presented in %, Fig. 2): BbF (12) > Chr (10) > BaP = BghiP = IndPir (8 each) > DahA (7) > BkF (6) > Ant = DahA (2 each) > Naf (1).

**Table 3:** The content of individual PAHs and their sum in samples (Me and RDS for *n* = 39).

No	Parameter	Minimum–Maximum	Median ± RSD
		[mg/kg]	
1	Naphthalene (Naf)	< 0.005–0.070	0.008 ± 0.016
2	Anthracene (Ant)	< 0005–0.883	0.016 ± 0.208
3	Chrysen (Chr)	0.006–5.630	0.069 ± 0.931
4	Benzo(a)anthracene (BaA)	< 0.005–4.800	0.044 ± 0.815
5	Dibenzo(a,h)anthracene (DahA)	< 0.005–1.100	0.020 ± 0.195
6	Benzo(a)pyrene (BaP)	< 0.005–5.880	0.058 ± 0.969
7	Benzo(b)fluoranthene (BbF)	0.006–6.340	0.092 ± 1.026
8	Benzo(k)fluoranthene (BkF)	< 0.005–2.920	0.041 ± 0.488
9	Benzo(g,h,i)perylene (BghiP)	0.006–4.320	0.068 ± 0.701
10	Indeno(1,2,3-c,d)pyrene (IndPir)	< 0.005–4.590	0.061 ± 0.750
Sum of PAHs		0.090–36.300	0.761 ± 6.691

The following substances were also found in the samples analysed: acenaphthene, benzo[a]fluoranthene, acenaphthylene, fluorene, phenanthrene, pyrene, benzo[e]pyrene. Their mean concentrations were 0.012, 0.332, 0.048, 0.019, 0.035, 0.095, 0.317 and 0.148, respectively (for *n* = *39*, in mg/kg). Due to the lack of regulatory limit values, the contamination level has not been evaluated for these compounds.

### Assessment of soil contamination with PAHs

The Regulation of the Minister of the Environment of 1 September 2016 on the method for conducting the assessment of land surface contamination^59^ was used as a basis for quantifying the contamination of soil with PAHs. This regulation specifies substances that are critical for the protection of the land surface and their permitted concentrations in the soil depending on the soil group as listed in the land register or the intended use of the land specified in the local spatial development plans (Table 4). The regulation specifies the permissible concentration of 10 PAHs: indeno[1,2,3-cd]pyrene, anthracene, chrysene, benzo[g,h,i]perylene, benzo[k]fluoranthene, benzo[b]fluoranthene, benzo[a]pyrene, dibenz[a,h]anthracene, benz[a]anthracene and naphthalene. The aforementioned regulation^59^ distinguishes four groups of land (I–IV) on the basis of its use. These are:

**Table 4:** Limit values for PAH content in soils according to Polish law (Regulation, 2016).

No	Parameter	Establish limit for				
		Agricultural areas	Forest areas	Unmanaged greenery	Managed greenery	Nature reserves
		[mg/kg]				
1	Naphthalene	0.1	1	0.1	0.1	0.1
2	Anthracene	0.2		0.2	0.2	0.2
3	Chrysen					
4	Benzo(a)anthracene	0.1		0.1	0.1	0.1
5	Dibenzo(a,h)anthracene					
6	Benzo(a)pyrene					
7	Benzo(b)fluoranthene					
8	Benzo(k)fluoranthene					
9	Benzo(g,h,i)perylene	0.2		0.2	0.2	0.2
10	Indeno(1,2,3-c,d)pyrene					

-Group I, which includes residential areas, built-up areas, urbanised undeveloped land or land under development, built-up agricultural land, recreational and leisure areas: leisure centres, children's playgrounds, beaches, organised parks, squares, green areas, sports areas such as stadiums, sports fields, ski jumping hills, toboggan runs, sports shooting ranges, swimming pools, golf courses and areas fulfilling entertainment functions such as funfairs, amusement parks, zoological and botanical gardens.

-Group II comprises: arable land and family allotments, orchards, meadows, pastures, pond bottoms, ditches.

**-**Group III includes: forests, wooded land and bushland, wooded land and bushland on agricultural land, wasteland, recreational and leisure areas, ecological sites.

-The last group IV includes industrial land, surface mining land, communication areas (roads, railway land), land intended for the construction of public roads or railway tracks.

For each of these four groups, limit values for risk-causing substances have been established, both for the 10 specified PAHs and for ΣPAH. These are (for the 0–0.25 cm b.g.l. layer; data in mg/kg DM):

-0.1 for Naf, BaA, DahA, BaP, BbF, and BkF (for land groups I and II),

-0.2 for Ant, Chr, BghiP, and IndPir (for land groups I and II),

-1 for all individual PAHs for land group III,

-20 for all individual PAHs for land group IV.

For ΣPAHs, these values are 30, 50, 300 and 3000 mg/kg DM, respectively.

**Figure 3:**
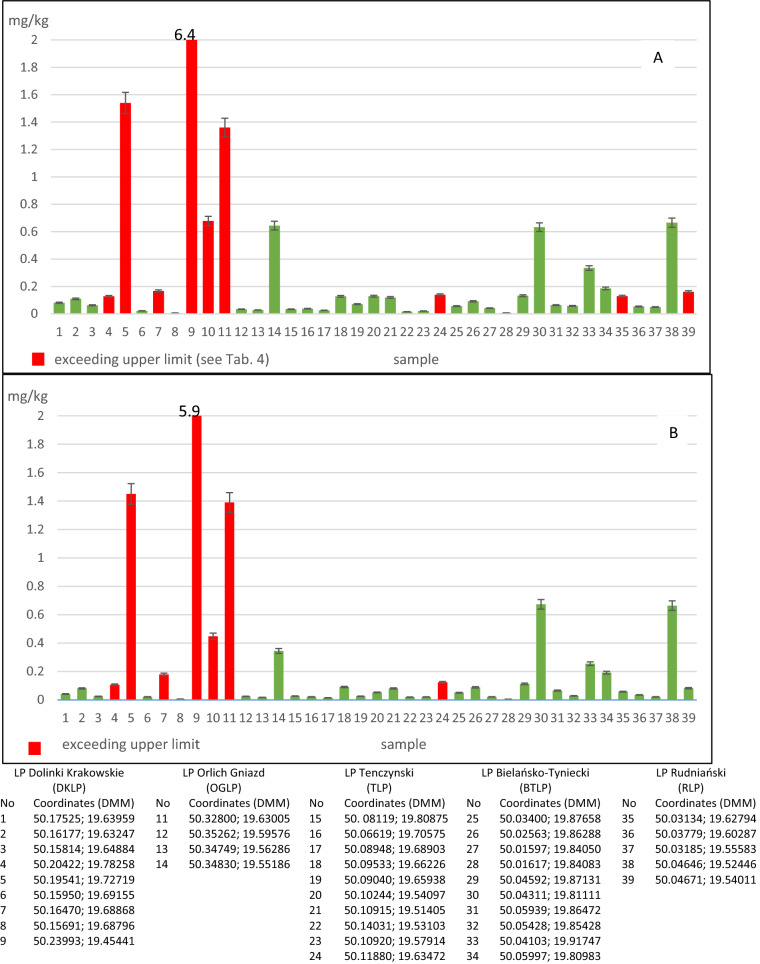
The content of benzo(b)fluoranthene (**A**) and benzo(a)pyrene (**B**) in collected samples.

When comparing the results obtained with limit values provided in the regulation^59^, it was found that the limits were exceeded in 37 cases (Table 5). The concentration of benzo[b]fluoranthene (Fig. 3A) and benzo[a]pyrene (Fig. 3B) exceeded the set limits to the greatest degree, while the concentrations of naphthalene and anthracene did not exceed the permissible levels.

**Table 5:** Exceeding the standards and increased PAH results in the tested samples.

L.p	Nazwa	Number of samples	
		Exceeding the limit	With higher value
1	Naphthalene	–	–
2	Anthracene	–	4
3	Chrysen	4	9
4	Benzo(a)anthracene	5	5
5	Dibenzo(a,h)anthracene	1	3
6	Benzo(a)pyrene	7	5
7	Benzo(b)fluoranthene	9	9
8	Benzo(k)fluoranthene	4	6
9	Benzo(g,h,i)perylene	3	9
10	Indeno(1,2,3-c,d)pyrene	4	7
Total		37	57

**Figure 4:**
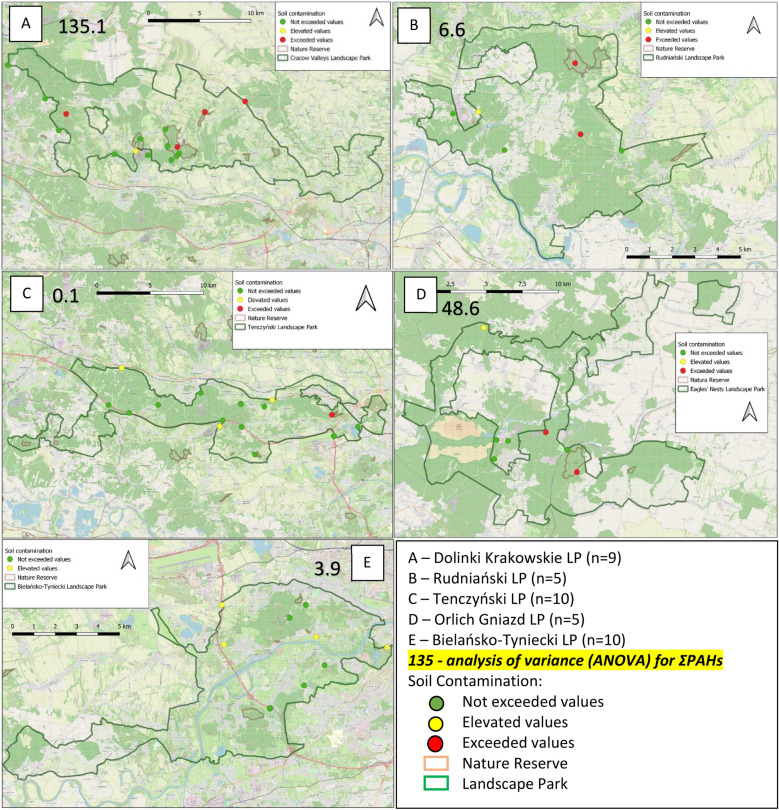
Soil PAH contamination on Małopolska Landscape Parks (OpenStreetMap).

The established limits were exceeded in 9 samples. Interestingly, these were collected from forest and agricultural areas located near transportation routes of various categories. In the Dolinki Krakowskie LP (Fig. 4A), the limits were exceeded in 4 samples and increased PAH concentrations were found in 1 sample. In the Rudniański LP (Fig. 4B), the limits were exceeded in 2 samples and increased PAH concentrations were found in 1 sample. In the Tenczyński LP (Fig. 4C), the limits were exceeded in 1 sample and increased PAH concentrations were found in 3 samples. In the Orlich Gniazd LP (Fig. 4D), the limits were exceeded in 2 samples and increased PAH concentrations were found in 1 sample. In the Bielańsko-Tyniecki LP (Fig. 4E), the limits were not exceeded in any of the samples, but increased PAH concentrations were found in 4 samples.

Increased PAH concentrations close to the established limits were found in 10 samples i.e., 57 determinations (Table 5). The majority of these results were obtained in the case of the samples collected in the Bielańsko-Tyniecki LP (Fig. 4E), mainly near transportation routes. It is worth noting that a considerable part of this landscape park is located inside a large city (Kraków), which may influence the results^60^. The greatest variation in the factors affecting ΣPAHs was found in the Dolinki Krakowskie LP: the calculated variance for the set of samples taken in this area was as high as 135.1, while for the Orlich Gniazd LP, the variance was 48.6. The variance calculated for the Rudniański LP was only 6.6, and for the Bielańsko-Tyniecki LP it was 3.9. The smallest variation in the factors affecting the occurrence of ΣPAHs in soils was found for the Tenczyński LP, the calculated variance for the analysed set of samples was only 0.1 (Fig. 4).

Comparing the ΣPAHs results obtained to the limits established in other countries^61,62^, it must be noted that according to the Dutch legislation^61^, almost 50% of the soil samples tested exceeded the permissible limit of 1 mg/kg. In contrast, none of the samples tested contained ΣPAHs above 40 mg/kg, which would indicate an exceedance of the “intervention” value. The Norwegian recommendations^62^ related to the permissible ΣPAHs content in soils in existing day-care centres and playgrounds are much stricter, recommending 2 mg/kg as the average safe guideline concentrations for a group of 10 samples taken, whereas for an individual sample this limit is set at 3 mg/kg. Applying these criteria, it should be noted that 18% of the samples analysed exceeded the 3 mg/kg value, especially those taken in the area of the Dolinki Krakowskie LP, Orlich Gniazd LP and Bielańsko-Tyniecki LP. In the case of the limit established by the Australian legislation^61^ for parks, recreational open space and playing fields, amounting to 40 mg/kg, it must be stated that it was not exceeded in any of the samples tested.

### Sources of PAHs

**Figure 5:**
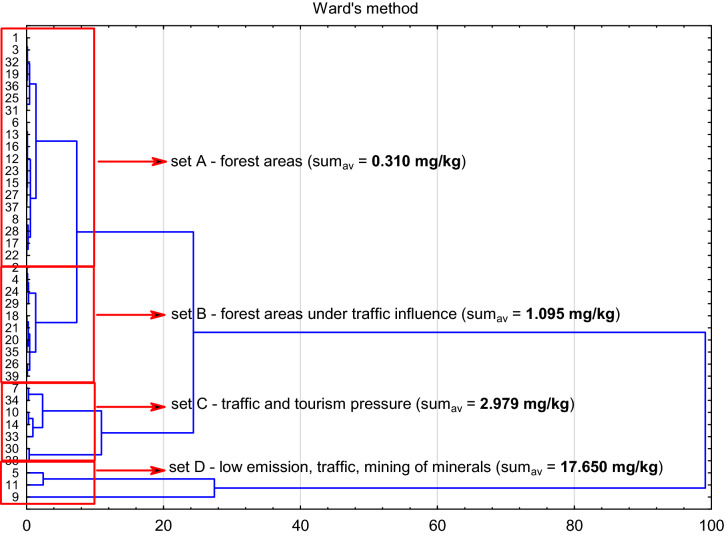
Cluster analysis for all tested samples (*n* = *39*) in terms of PAH content.

Bojakowska and Sokołowska^55^ state that the composition of the PAHs mix in soils near emitters such as industrial plants varies and may be related to the composition of materials being processed at these sites. Increased or excessive PAHs concentrations in the soils analysed do not occur in the vicinity of major industrial plants located near the boundaries of individual landscape parks. Lower concentrations of PAHs were found in soil samples collected in forest areas that are not affected by other factors (set A, Fig. 5). The mean concentration of all PAHs for this set amounted to 0.310 mg/kg.

Increased concentrations of PAHs and concentrations of PAHs exceeding the specified limits occurred mostly in the case of soil samples collected in the vicinity of transportation routes (set B, Fig. 5). The mean PAH concentration for this group of samples was 1.095 mg/kg. A considerably higher mean concentration of PAHs (amounting to 2.979 mg/kg) was found in soil samples collected in locations that are affected by tourist traffic and transport (set C, Fig. 5). The highest PAH concentrations were found in soils that are affected by low emissions, by excavation plants i.e. quarries, especially the quarry located inside the Orlich Gniazd LP (sample G-PKOG-02, Fig. 5) and by roads. Interestingly, these higher concentrations were mostly related to the impact of province, commune and local roads (set D, Fig. 5), while permissible PAH concentrations were not exceeded in samples collected near national roads and a motorway with very heavy traffic. This may be related to the higher speeds of vehicles travelling along the latter roads, fewer opportunities for them to stop and fewer collisions which can cause spills of various substances into the soil^21–25,36,63^.

**Figure 6:**
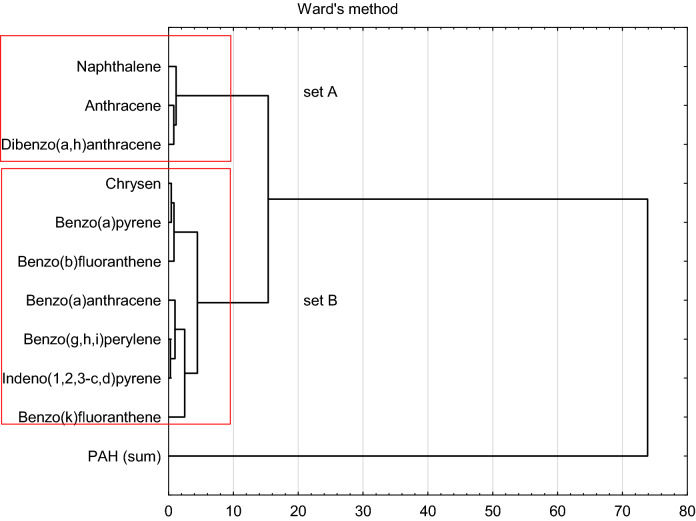
Cluster analysis for the tested samples in terms of the distribution of individual hydrocarbons.

The most prevalent compounds found in samples collected near roadways were benzo[b]fluoranthene and chrysene. Moreover, the concentrations of indeno[1,2,3-cd]pyrene and benzo[g,h,i]perylene were higher than in the case of soil samples collected further away from roadways or outside areas affected by transportation routes (set B, Fig. 6). Soils near motorways were characterised by higher concentrations of benzo[g,h,i]perylene, which may indicate the presence of substances from the combustion of fuels, the wear of road surface and vehicle tyres and motor oil spills^57,64–66^. Worryingly, permissible values were exceeded in the case of samples collected in four out of five nature reserves located in Małopolska (Table 6). The samples were collected close to transportation routes (about 1.5 m from the roadway) passing through nature reserves or along their boundaries. Only in the case of Dolina Racławki Nature Reserve was the sample unaffected by the transportation route. Instead, the possible reason for high PAH concentrations found there is the transport of pollutants by the Racławka Stream, which occurs especially during freshets, causing the deposition of pollutants in fluvial terraces. The role of fluvial deposits as sources of PAH contamination in fluvial terraces has been discussed by Gocht^67^.

**Table 6:** PAH content in soils collected in nature reserves in Malopolska region.

Nature Reserve	Landscaped park	Sample number of exceeded of individual PAHs limit	Ingredient exceeded	Traffic influence
Rudno Stream Valley	Rudniański	1	Benzo(b)fluoranthene	yes
Szklarka Valley	Dolinki Krakowskie	7	Chrysen Benzo(a)anthracene Benzo(a)pyrene Benzo(b)fluoranthene Benzo(k)fluoranthene Benzo(g,h,i)perylene Indeno(1,2,3-c,d)pyrene	yes
Pazurek	Orlich Gniazd	7	Chrysen Benzo(a)anthracene Benzo(a)pyrene Benzo(b)fluoranthene Benzo(k)fluoranthene Benzo(g,h,i)perylene Indeno(1,2,3-c,d)pyrene	yes
Racławka Valley	Dolinki Krakowskie	3	Benzo(a)anthracene Benzo(a)pyrene Benzo(b)fluoranthene	no
Eliaszówka Valley	Dolinki Krakowskie	–	–	yes

Finally, it is worth looking at the structure of the Polish regulation pertaining to the pollution of soil in the context of PAH contamination assessment. When classifying areas from which soil samples are taken, the intended land use specified in the local spatial development plans, or the land “utility”, is taken into consideration. Such a classification method can affect the results of contamination assessment, especially given the impact of various land development forms (such as industrial plants or transport routes etc.) on neighbouring areas which often have significant natural value. However, only the most restrictive forms of protection, i.e. nature reserves and national parks, are assigned category II regardless of intended use or its current character.

## Conclusions

The present study has demonstrated varying concentrations of PAHs in soils near different sources of pollution and in different locations across protected areas. This allowed the following conclusions to be drawn:

- the concentration of PAHs in the soils of the landscape parks studied is usually low; however, in a few cases, the permissible concentrations were exceeded, sometimes substantially;
- permissible concentration levels were exceeded in the case of 10% of determinations, and occurred in 9 out of 39 samples analysed;
- increased concentration levels were found in the case of 14.61% of all results obtained and occurred in another 10 samples;
- the sum of the ΣPAHs concentration limit was not exceeded in any of the samples;
- it is worth considering increasing the classification category for the most valuable natural areas in Poland (e.g. nature reserves) in the existing regulations;
- it is also worth considering assigning a higher classification category to the remaining forms of nature protection, including e.g. landscape parks.

### Recommendations

In the case of carcinogenic pollutants, it is necessary to create more detailed regulations in the Polish regulatory system, taking into consideration the hazards related to potential and actual pollution of soils (covering e.g. contamination with PAHs), as well as to conduct continuous monitoring, especially near potential pollution emitters. It is also worth considering implementing a new system of pollution assessment, especially one that would enable area classification taking into account the neighbouring areas and the impact of pollutants on these areas.

In addition to improved regulations, it is also important to enforce them, especially when it comes to continuous monitoring conducted by the authorities (Province Inspectorates of Environmental Protection, Regional Directorates for Environmental Protection, Landscape Park Services). Considering the proven negative impact of polycyclic aromatic hydrocarbons on human health (and probably also on other elements of the environment), it is also required to reduce the highest contamination levels by means of soil remediation or phytoremediation as well as a reduction in emissions.

## Data Availability

The datasets used and/or analysed during the current study are available from the corresponding author upon reasonable request.
